# Effect of the area of a lithium niobate transducer on the efficiency of ultrasonic atomization driven by resonance vibration

**DOI:** 10.1016/j.ultsonch.2022.106019

**Published:** 2022-04-28

**Authors:** Keisuke Yoshioka, Yuta Kurashina, Ami Ogawa, Takumi Asakura

**Affiliations:** a School of Mechanical Engineering, Graduate School of Science and Technology, Tokyo University of Science, Japan; b Department of Materials Science and Engineering, School of Materials and Chemical Technology, Tokyo Institute of Technology, Japan; c Department of Mechanical Systems Engineering, Faculty of Engineering, Tokyo University of Agriculture and Technology, Japan; d Department of System Design Engineering, Faculty of Science and Technology, Keio University, Yokohama, Japan

**Keywords:** Ultrasonic atomization, Lithium niobate device, Drug nebulization, Atomization efficiency, Vibration mode, Wearable humidifier

## Abstract

In recent years, individual control of one’s personal environment has been drawing increasing attention due to the growing interest in health care. Wearable devices are especially useful because of their controllability regardless of location. Humidity is one of the inevitable factors in the personal environment as a preventive against infectious diseases. Although atomization devices are commonly used as a method of humidity control, at present, there are no wearable humidity control devices. Vibration of a lithium niobate (LN) device in the thickness mode is a promising piezoelectric method for miniaturization of atomization devices for humidity control. To miniaturize the atomization device, the transducer size needs to be small not so much as to decrease the atomization efficiency. However, the effect of the device area on the atomization efficiency of LN at a size suitable for mounting in wearable devices has not been studied. Here, we conducted an atomization demonstration of LN devices with different sizes to evaluate particle size and atomization efficiency. Furthermore, to reveal the relationship between vibration behavior and atomization efficiency, resonance vibration in the MHz frequency band was evaluated by the finite element method and an impedance analyzer. The results showed that the peak size of water particles atomized by each device was in the range of 3.2 to 4.2 µm, which is smaller than particles produced by typical piezoelectric ceramics. Moreover, the best LN size for efficient atomization was found to be 8 mm × 10 mm among the five LN device sizes used in experiments. From the relationship between vibration behavior and atomization efficiency, the size of the transducer was suggested to affect the vibration mode. The obtained result suggested that the LN device is suitable for small wearable nebulizer devices.

## Highlights

• Each LN-based device produced peak water particle sizes in the range 3.2–4.2 µm.
• Larger transducer area increased atomization volume but decreased efficiency.
• The optimal LN size for efficient atomization area was found to be 8 mm × 10 mm.
• FEM results and impedance analysis suggested that mode mixing reduced atomization.

## 1 Introduction

The fight against infectious diseases, such as the recent COVID-19 pandemic [1], is one of the primary medical concerns in the history of humankind [2]. One popular and important countermeasure is to establish a humidified space on a daily basis to deactivate the virus [3]. For airborne infections, the nebulization of liquid drugs to treat respiratory diseases is also effective [4]. From these observations, the study of atomization contributes to the prevention of infectious diseases. Ultrasonic atomization [5] is one of the most widely-used atomization technologies. Ultrasonic atomization is generally applied to atomizing products in general living spaces [6] and to devices in otolaryngological treatment [7]. These ultrasonic atomizers are employed to humidify the entire space [8] and inhale the atomized agent [9]; however, most ultrasonic atomizers have a limited atomization area depending on the installation location. Regardless, individual control of each personal environment unconstrained by location has been drawing increasing attention over recent years due to the growing interest in health care [10], [11]. In the current situation of the recent pandemic caused by infectious diseases, the provision of a humid environment suited for each individual and self-medication by nebulization is needed. Therefore, a portable atomizer with high efficiency and compact size is indispensable to achieve a sufficiently humid environment on a personal scale.

To improve the efficiency and miniaturization of the atomizer, considerations for the ultrasonic transducers are important. Currently, the piezoelectric ceramic lead zirconate titanate (PZT) is commonly used as an atomizing transducer because PZT has a large electromechanical coupling coefficient [12]. In addition to PZT, lithium niobate (LN) is also known as a piezoelectric material. The efficiency of LN is generally lower than that of PZT because it has a small electromechanical coupling coefficient [13]. Meanwhile, the efficiency of LN surpasses that of PZT in terms of the performance index because the quality factor in the thickness mode was found to be much higher than that for PZT among the vibration modes of both LN and PZT [14], [15]. Additionally, the thickness mode of LN has a faster wave propagation speed and much larger maximum amplitude displacement than vibrations in PZT [16]. Furthermore, LN has a higher Young’s modulus because it has a greater mechanical hardness than PZT [17], [18].

Therefore, LN is widely used as a high-frequency surface acoustic wave (SAW) device because its high stiffness leads to a higher resonance frequency than that for PZT [19], [20]. Thus, recent studies have shown that LN devices vibrating in the thickness mode are more efficient than PZT vibration with respect to atomization. By applying LN as the transducer in an atomizer, atomization is expected to be highly efficient compared to conventional PZT-based atomizers. For the practical application of atomizers made with LN, it is necessary to determine a suitable size of the LN transducer. However, the efficiency of LN per unit area and the effect of the vibration mode on the efficiency have not yet been clarified.

Here, we demonstrate the atomization behavior of LN devices with five different sizes and evaluate their efficiency in each of the resonance modes. The particle sizes of atomized water were measured, and the atomization efficiency of each device was determined based on the atomization area and power consumption. In addition, the vibration modes around the thickness mode were calculated by the finite element method (FEM) to visualize how the vibration of the device promotes atomization.

## 2 Materials and methods

### 2.1 Preparation of the LN device

To investigate the effect of the device size on the atomization characteristics, five LN devices with different sizes (=4 × 5 mm, 8 × 10 mm, 12 × 15 mm, 16 × 20 mm, and 20 × 25 mm) were prepared. The 127.68° Y-rotated cut has long been known to provide superior mechanical properties in terms of coupling and especially in suppressing wasteful spurious modes [21]. Thereby, a LN plate with a 127.86° Y-rotated cut (Yamajyu Ceramics, Aichi, Japan) was used. The thickness of these five devices was 0.5 mm, and the area was changed while maintaining a constant aspect ratio of 4:5. To vibrate efficiently without breakage, the thickness of the LN device was determined to be 0.5 mm based on previous research [22], [23]. The device was cut into a rectangular shape to allow it to be held with pins at the edges during the experiment. The top and bottom surfaces of the LN device were processed to deposit 100-nm-thick Au to provide conductors to energize the device from the top and bottom surfaces during atomization and use it as an atomization transducer. To use the entire LN as a transducer by applying a voltage in the thickness direction, Au was deposited on the entire surface to form an electrode. To efficiently drive the LN devices, Au thickness of the LN device was determined to be 100 nm based on previous research [24], [25]. Furthermore, LN devices were also processed for oxygen reduction treatment. Thus, the resistivity of the LN device was decreased by reducing the oxygen content.

### 2.2 Devices fabrication

The experimental system for ultrasonic atomization consisted of a signal generator (FGX-2220, TEXIO, Kanagawa, Japan), a voltage amplifier (LZY-22+, Mini-Circuits, Brooklyn, NY, USA), and an atomization point. In the experiment, the frequency and voltage were input by a signal generator, the voltage was amplified by a voltage amplifier connected to the signal generator, and the amplified signal was output to the atomization point. The atomization point consisted of an LN device sandwiched between a steel plate and a probe fabricated by a 3D printer. A conductive pin was attached to the tip of the probe, and the pin was connected to the voltage amplifier by a lead wire. The other electrode was connected to a steel plate for grounding. Hence, voltage was applied to the top surface of the LN device via the pin. Furthermore, after soldering the conductor to the steel plate, the signal was output to the LN device by energizing the probe and the steel plate from the top and bottom surfaces during the experiment.

### 2.3 Observation of droplets using a high-speed camera

**Fig. 1:**
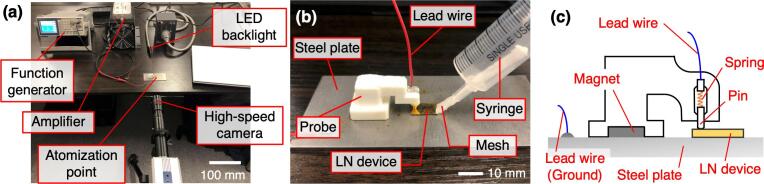
Experimental setup for observation and evaluation of water particles atomized by LN device. (a) Atomized water was illuminated by LED backlighting and photographed by a high-speed camera. Water was dropped from a pipette and observed immediately after atomization. An AC voltage was supplied between the probe holding the LN device and the steel plate. (b) In experiments to evaluate the efficiency of the atomization, a mesh (wick) was attached to the syringe to provide a continuous supply of water. (c) A conductive pin is attached to the tip of the probe to hold the LN device. In addition, a spring is attached to the inside of the pin, which does not interfere with the vibration of the LN device.

To observe ultrasonic atomization, an experimental system was set up as shown in Fig. 1(a) and the droplets were imaged with a high-speed camera (HAS-D73, DHITECT, Tokyo, Japan). During the shooting, white light from the LED light source (UFLS-75, U-TECHNOLOGY, Tokyo, Japan) was directed through the droplets to the high-speed camera. The experimental procedure was as follows. A signal generator produced a sinusoidal signal at the resonance frequency via a voltage amplifier to drive the LN device while the drops were imaged using a high-speed camera. In the experiment, 1.2 V_pp_ was applied and common tap water was used as the liquid to be atomized. Furthermore, each LN device was driven at around 7 MHz (7.38 MHz for a size of 4 × 5 mm, 7.14 MHz for 8 × 10 mm, 7.12 MHz for 12 × 15 mm, 6.91 MHz for 16 × 20 mm, and 6.90 MHz for 20 × 25 mm). The atomized mist was magnified and imaged using a high-speed camera. When observing the atomization particles, the shooting speed and shutter speed of the camera were set to 2,000 fps and 1/200,000 s, respectively. Then, after observing the atomization particles, the image analysis software (ImageJ, ver. 1.53 m, National Institutes of Health, Bethesda, MD, USA) was used to analyze the particle size based on the captured images. Next, the overall atomization phenomenon was imaged using a high-speed camera mated to a wide-angle lens (AT-X PRO D, KenkoTokina, Tokyo, Japan). When observing the atomization phenomenon, the shooting speed and shutter speed were set to 2,000 fps and 1/10,000 s, respectively. Additionally, the particle size was analyzed with biocompatible solutions with various characteristics, assuming that the solutions are used for medical applications. In particular, liquid drugs around 1.0 cp are commonly used in nebulizer treatment [26], [27], but experiments were conducted assuming various viscosities of liquid. Phosphate buffered saline (PBS; FUJIFILM Wako Pure Chemical Corporation, Osaka, Japan) and glycerol (FUJIFILM Wako Pure Chemical Corporation, Osaka, Japan) were used as solutions. The viscosity of PBS (≈1.0 cp [28]) is almost the same as that of water, whereas the viscosity of glycerol solutions is easily modified by adjusting the concentration [29]. Hence, three various viscosities of glycerol (≈10, 30 and 100 cp) were prepared. Note that, the 8 × 10 mm LN device was used as a representative transducer for atomization of these solutions.

### 2.4 Measurement of atomization volume

To quantify the atomizing capacity, the effect of the LN device size on the atomization volume was evaluated. In addition, the power consumption of each device during atomization was measured. To realize a continuous water supply and atomization at the atomization point, we created a device to supply water to the surface of the transducer by attaching a free-flow wick to a syringe [Fig. 1(b)]. During the experiment, the water was supplied through a mesh contacting the edge of the transducer, and the weight change of the water in the syringe was measured by an electronic balance (KD-321, TANITA, Tokyo, Japan). To measure the power consumption of the LN device during atomization, the atomization point and an oscilloscope (DS-5654A, IWATSU, Tokyo, Japan) were connected by a voltage probe (SS-101R, IWATSU) and a current probe (CT2, Tektronix, Beaverton, OR, USA). The current and voltage flowing through the LN device were measured by these probes.

### 2.5 Numerical analysis of resonance modes

To analyze the vibration behavior of each LN device during atomization, the FEM was used to analyze the vibration modes. Specifically, an eigenvalue analysis was used to evaluate the relationship between the resonance frequencies and mode shapes. Three device sizes of 4 × 5 mm, 8 × 10 mm, and 20 × 25 mm, were analyzed and compared. The physical properties of the LN [30] were adopted as the material conditions, and external loads and constraints were not defined for boundary conditions. Then, the LN plate was simulated as a free-boundary plate. The number of discrete mesh elements for the LN devices with sizes of 4 × 5 mm, 8 × 10 mm, and 20 × 25 mm was 6913, 12,523, and 26,223, respectively, depending on the size of the LN device. Moreover, the surface center and edge elements of the analytical structure were selected for measurement of the amplitude because the droplet was placed between the center and the edge of the LN device. The edge element was unified as an element in the x-axis direction from the surface center element. Note that the vertical axis in the amplitude analysis was converted to a common logarithm scale and multiplied by 10. Furthermore, the maximum value of the converted data was normalized to 0 dB.

### 2.6 Frequency response measurement of LN devices

To evaluate the resonance frequency of the LN devices, the frequency response of each device was measured with a frequency response analyzer (FRA5097, NF, Kanagawa, Japan). The frequency was swept from 4 to 8 MHz at 5-kHz intervals. Note that the applied voltage was set to 1 V.

## 3 Results and discussion

### 3.1 Particle sizes of water atomized by the LN device

**Fig. 2:**
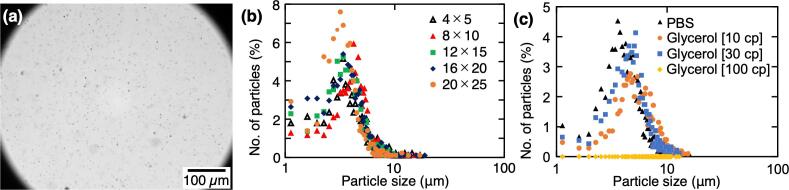
Measurement of water particles atomized by LN device. (a) Example image of water particles atomized using a 12 mm × 15 mm LN device and photographed by a high-speed camera. (b) Relationship between the number of particles and the water particle size atomized by each device (*n* = 850–900). (c) Relationship between the number of particles and the particle size atomized by various viscosity liquids (*n* = 900–1000).

The appearance of the water particles atomized by the five LN devices with the various device areas was observed by the high-speed camera [Fig. 2(a)]. The water particle size was measured from the captured movies [Fig. 2(b)]. This analysis revealed that the peak size of the water particles atomized by each device was in the range of 3.2 to 4.2 µm. In all device series, 90% of the particles were within a range of 10 µm. This indicates that the water particle size is almost the same, independent of the size of the LN device.

Particle size is extremely important for the nebulization of liquid drugs. In comparison, the water particles atomized by the five types of LN devices were smaller than those generated by typical PZT devices, which produced water particles with a diameter of around 7 µm [31], [32]. Furthermore, to compare studies with the PZT and LN, the materials of the atomization device, the driving frequency, the particle peak size, solutions and the vibration mode are summarized (Table 1) [33], [34], [35], [36]. This indicates that LN devices are better suited to produce smaller particles than PZT. The method used in this study, in which Au is coated on both sides of LN, is particularly suitable because it is easy to fabricate and does not require microfabrication, as with the SAW devices. To apply the transducer to the nebulization of liquid drugs, the liquid drug must be able to reach the lower respiratory tract, which is greatly influenced by the particle size [37], [38], [39], [40]. From previous research, water particle sizes of 2–6 µm are considered suitable for inhaling bronchodilators or steroids [37]. For infants and young children with small airway diameters, smaller water particles are considered beneficial [38]. Furthermore, particles with a diameter greater than 5 µm have a greater chance to be trapped in the throat by inertial impaction; meanwhile, particles within the 1- to 5-µm range are deposited deeply into the lungs by sedimentation [39], [40]. In other words, the water particles atomized by the LN device are more suitable for the nebulization of liquid drugs compared with conventional PZT devices. From the atomization experiments, 10 cp glycerol, and 30 cp glycerol were atomized, meanwhile 100 cp glycerol could not atomize. Relationship between the number of particles and the particle size atomized by various solutions is shown in Fig. 2(c). This indicated that the peak size of the particles atomized with various viscosities was to be 3.6 μm for PBS, 4.7 μm for 10 cp glycerol, and 5.2 μm for 30 cp glycerol. The peak of the particle size of PBS, whose viscosity was almost the same as that of water, was close to that of water, while the peak of the particle size of glycerol solution increased with increasing viscosity. Therefore, the viscosity of the solution affects the particle size. From our results, atomized particles generated by liquids of 10 cp or less can be reached to the lungs. Although, atomization of highly viscous drug liquids is not considered suitable for infants or for the treatment of pulmonary diseases.

**Table 1:** Comparison of solutions atomized by each ultrasonic device.

Device material	Driving frequency [MHz]	Particle peak size [μm]	Solution	Vibration mode	References
PZT	0.5	7∼8	Water	Thickness	Y.- L. Song *et al.* 2020 [33]
PZT	1.6	6∼8	Myoglobin	Thickness	C. T. Pan *et al.* 2007 [34]
LN	7	3.2∼4.2	Water	Thickness	*This research*
LN	20	3.95	Salbutamol / octanol	SAW	A. Qi *et al.* 2009 [35]
LN	48	3∼4	Water	SAW	A. G. Niam *et al.* 2020 [36]

### 3.2 Relationship between atomization volume and power consumption of LN device

**Fig. 3:**
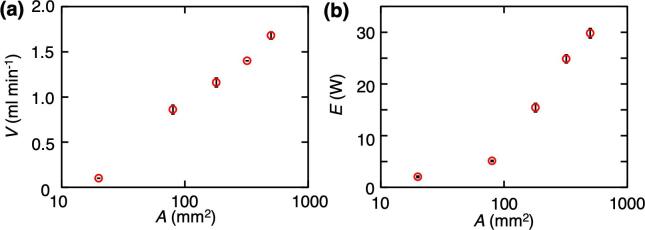
Measurement of atomized volume and power consumption for various atomization areas. (a) Relationship between the atomization area of each LN device, *A*, and the atomized volume, *V*. (b) Relationship between *A* and the electricity consumption, *E* (*n* = 5, mean ± SD).

The relationship between the atomization area of each LN device, *A*, and the atomized volume, *V*, was evaluated [Fig. 3(a)]. The results indicate that the atomized volume increased gradually as the area of the device increased. The power consumption of the device also increased as the area increased. For ultrasonic atomization devices with PZT transducers, the atomized volume reportedly increased because the higher the applied voltage, the larger the amplitude displacement jof the transducer via the reverse piezoelectric effect [41]. In our device, the amplitude displacement of the device increased as the area of the LN transducer increased, and the atomization volume increased [Fig. 3(a)]. The relationship between *A* and the power consumption, *E*, was also evaluated [Fig. 3(b)]. Considering the atomization area of the LN device on a log scale, the relationship between *A* and *V* was linear, while the relationship between *A* and *E* increased exponentially. According to these results, the relationship between the atomization volume and the power consumption is close.

### 3.3 Atomization efficiency of the LN device

**Fig. 4:**
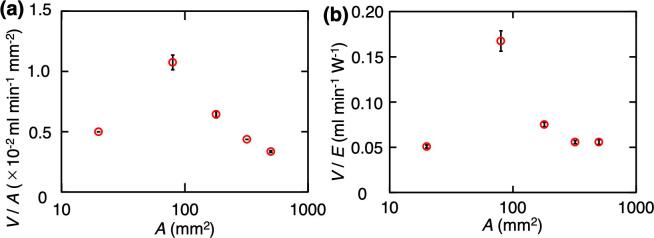
Evaluation of atomization efficiency for various atomization areas. (a) Relationship between *A* and *V*/*A*. (b) Relationship between *A* and *V*/*E* (*n* = 5, mean ± SD).

The atomization efficiency was evaluated based on the measurement results for atomization volume [Fig. 3(a)] and power consumption [Fig. 3(b)]. The atomization efficiency (Fig. 4) is expressed as the amount of atomization per device area (*V*/*A*), and the atomization volume per unit of power consumption (*V*/*E*). The relationship between *A* and *V*/*A* [Fig. 4(a)] shows a peak (=1.1 × 10^-2^ mL min^−1^ mm^−2^) at *A* = 80 mm^2^, although *V* tended to increase as *A* increased [Fig. 3(a)]. Moreover, the relationship between *A* and *A*/*E* [Fig. 4(b)] has also a peak (=0.17 mL min^−1^ W^−1^) at the same value of *A*, although *V* tended to increase as *E* increased [Fig. 3(b)]. According to these results, the existence of an optimum device area for the application of LN transducers to atomization devices was suggested. From the results of this study, the most efficient size of the atomization area is 8 × 10 mm. Because a device this size is smaller than commonly used PZT transducers (∼100π mm^2^) [42], [43], this size is suitable for the miniaturization required for wearables. For example, a palm-sized wearable device is being developed [44], [45]. The size of the LN device derived in this research is small enough to be mounted in a palm-sized device.

### 3.4 Observation of water atomization immediately after the start of the resonance vibration

**Fig. 5:**
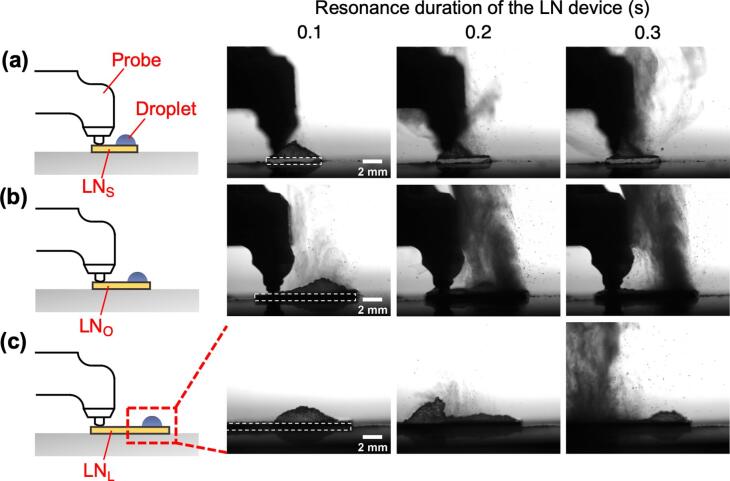
High-speed camera images of start of water atomization by three LN device sizes: (a) the smallest LN device at 4 × 5 mm (LN_S_), (b) the optimal LN device at 8 × 10 mm (LN_O_), and (c) the largest LN device at 20 × 25 mm (LN_L_). The location of the LN device in each image set is indicated by a dashed line 0.1 s after the start of the vibration.

To evaluate the onset of atomization, the water atomization immediately after the start of the resonance vibration was observed with a wide-angle lens (AT-X M100 PRO D, Kenko Tokina). The following devices were compared: the smallest LN device with an area of 4 × 5 mm (LN_S_), the optimal LN device with an area of 8 × 10 mm (LN_O_), and the largest LN device with an area of 20 × 25 mm (LN_L_) to observe the salient characteristics (Fig. 5). With LN_S_ and LN_L_, the surface of the droplet vibrated 0.1 s after the start of actuation. The surface of the droplet began to vibrate even more intensely, and the water particles began to be atomized 0.2 s after the start of vibration. Finally, atomization from the droplet was steady 0.3 s after the start of actuation. Meanwhile, in the LN_O_, the water particles already began to atomize 0.1 s after the start of vibration. Then, droplet atomization from the LN_O_ was steady 0.2 s after the start of vibration. These results indicate that the time until the start of atomization was short with the optimal LN device. This difference is considered due to the vibration being excited on the LN device, which is also related to the atomization efficiency.

### 3.5 Vibration mode of the LN device during atomization

**Fig. 6:**
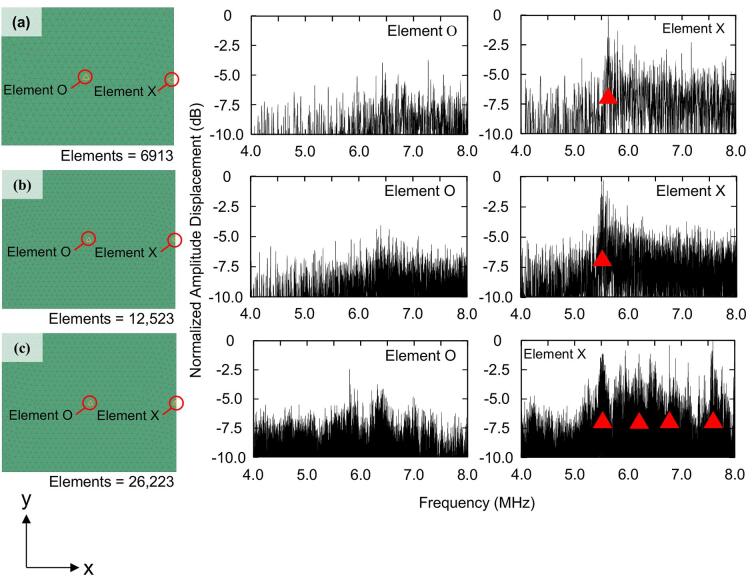
Relationship between frequency and displacement at element O and element X for LN devices from 4 MHz to 8 MHz. The vertical axis is normalized to the displacement with respect to 0 dB. The amplitude peaks of (a) LN_S_, (b) LN_O_, and (c) LN_L_ are indicated by red arrows.

The experiments on atomization efficiency (Fig. 4) identified a difference in the atomization efficiency that depends on the area of the LN transducer. Although an increase in the area clearly results in an increased atomization volume, the decrease in atomization efficiency beyond the peak is questionable. Therefore, the vibration behavior of LN_S_, LN_O_, and LN_L_ during atomization were analyzed. Here, an FEM analysis was used to evaluate the relationship between the resonance frequency and the deformation of a mesh element in the transducer. From the analysis results (Fig. 6), the element X of LN_S_ and LN_O_ has a resonance frequency with a large amplitude converging around 5.5 MHz [red arrows in Fig. 6(a,b)], while element X of LN_L_ has discrete resonance frequencies [red arrows in Fig. 6(c)]. A possible explanation for the decrease in atomization efficiency with increasing atomization area is the lack of convergence of the amplitude peaks. This means that the amplitude peaks dispersed at different frequencies simulated in the FEM analysis should be superimposed in the actual vibration to decrease the vibration efficiency.

**Fig. 7:**
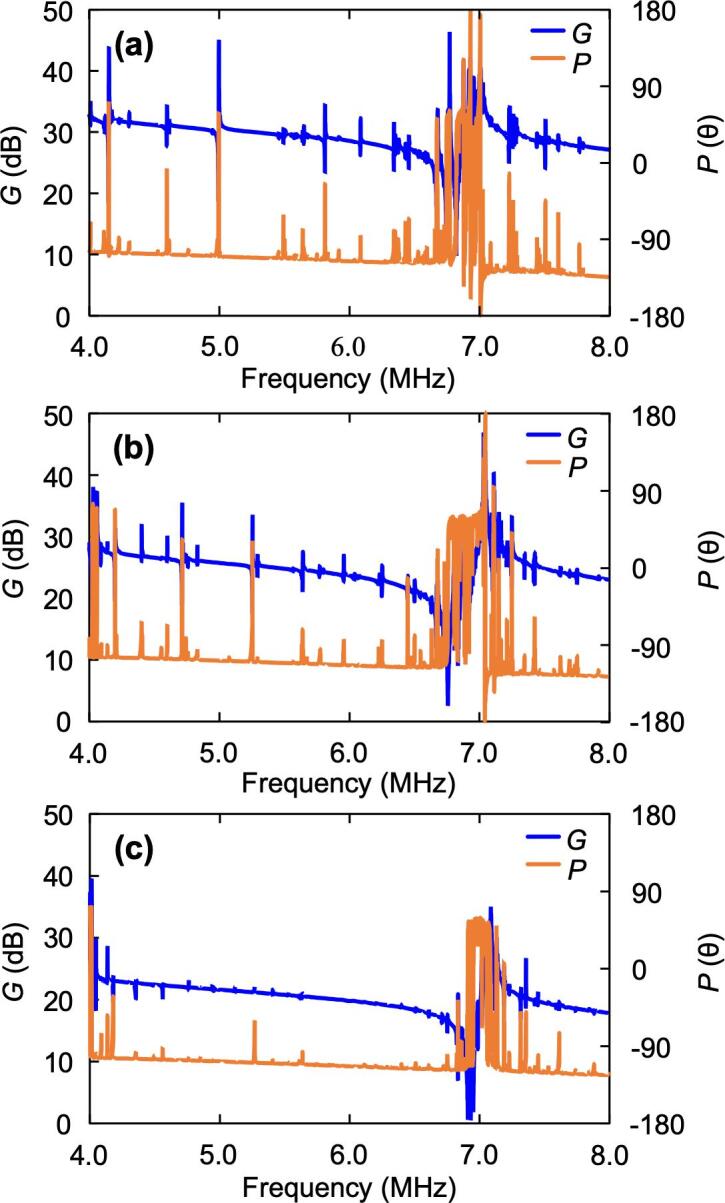
Frequency response of each device from 4 MHz to 8 MHz. Phase, *P*, and gain, *G*, of (a) LN_S_, (b) LN_O_, and (c) LN_L_ are shown on the vertical axis. The gain on the vertical axis is indicated by a log scale, and the units are converted to dB.

Here, the actual resonance frequency was measured by a frequency response analyzer (Fig. 7). Around 7 MHz, where atomization was observed, the peaks in the LN_L_ phase were discrete and the width of the phase change was small. Conversely, the phase change for LN_S_ around 7 MHz was large; however it was discretely distributed, and the vibration energy was dispersed around the frequency region. In contrast, the phase change of LN_O_ was focused on a single frequency at 7 MHz, and the extent of phase change was large. Although the resonance frequencies were different between the measurement and FEM results because the boundary conditions were not completely satisfied, the characteristics of convergence and divergence of the amplitude peaks in the FEM analysis were also observed in the measurement of the frequency response. Therefore, the larger the size of the atomization area, the higher the atomization volume, but the transmission efficiency of the vibration decreased due to the mixing of modes. These results suggest that LN_O_ led to the optimal atomization efficiency among the prepared atomization sizes.

## 4 Conclusion

In this study, the effect of the LN transducer area on ultrasonic atomization was evaluated. The atomization volume increased with increasing transducer area, but the atomization efficiency decreased beyond a certain area increase. This was due to the LN device size affecting the vibration mode. Accordingly, the results of this study provide significant guidelines for determining the appropriate LN size when incorporating humidity functions into wearable devices. These wearable applications for small nebulizers and humidifiers will lead to improved living environments and human health in the medical and built-environment fields, contributing to the further development of design engineering, medicine, and public health.

## Funding

This work was supported in part by the 10.13039/100008732Uehara Memorial Foundation (Tokyo, Japan).

## Declaration of Competing Interest

The authors declare that they have no known competing financial interests or personal relationships that could have appeared to influence the work reported in this paper.
